# First-in-Human Side-to-Side Magnetic Compression Duodeno-ileostomy with the Magnet Anastomosis System

**DOI:** 10.1007/s11695-023-06708-x

**Published:** 2023-07-02

**Authors:** Michel Gagner, David Abuladze, Levan Koiava, J. N. Buchwald, Nathalie Van Sante, Todd Krinke

**Affiliations:** 1 Department of Surgery, Westmount Square Surgical Center, 1 Westmount Square, Suite 801, Westmount, QC H3Z2P9 Canada; 2 Department of General & Bariatric Surgery, Innova Medical Center, Tbilisi, Republic of Georgia; 3 Division of Scientific Research Writing, Medwrite, Maiden Rock, WI USA; 4 NVS Consulting, Brussels, Belgium; 5 GT Metabolic Solutions, San Jose, CA USA

**Keywords:** Metabolic/bariatric surgery, Magnetic compression anastomosis, Duodeno-ileostomy, Sleeve gastrectomy, Obesity, Type 2 diabetes

## Abstract

**Purposes:**

Classical gastrointestinal anastomoses are formed with sutures and/or metal staples, resulting in significant bleeding and leak rates. This study evaluated the feasibility and safety of the novel magnet anastomosis system (MS) to create a side-to-side duodeno-ileal (DI) diversion for weight loss and type 2 diabetes (T2D) resolution.

**Materials and Methods:**

Patients with severe obesity (body mass index (BMI) ≥ 35 kg/m^2^ with/without T2D (HbA1_C_ ≥ 6.5%)) underwent the study procedure, a side-to-side MS DI diversion, with a standard sleeve gastrectomy (SG). A linear magnet was delivered by flexible endoscopy to a point 250 cm proximal to the ileocecal valve; a second magnet was positioned in the first part of the duodenum; the bowel segments containing magnets were apposed, initiating gradual anastomosis formation. Laparoscopic assistance was used to obtain bowel measurements, obviate tissue interposition, and close mesenteric defects.

**Results:**

Between November 22 and 26, 2021, 5 female patients (mean weight 117.6 ± 7.1 kg, BMI (kg/m^2^) 44.4 ± 2.2) underwent side-to-side MS DI + SG. All magnets were successfully placed, expelled without re-intervention, and formed patent durable anastomoses. Total weight loss at 12 months was 34.0 ± 1.4% (SEM); excess weight loss, 80.2 ± 6.6%; and BMI reduction, 15.1. Mean HbA1_C_ (%) dropped from 6.8 ± 0.8 to 4.8 ± 0.2; and glucose (mg/dL), from 134.3 ± 17.9 to 87.3 ± 6.3 (mean reduction, 47.0 mg/dL). There was no anastomotic bleeding, leakage, obstruction, or infection and no mortality.

**Conclusions:**

Creation of a side-to-side magnetic compression anastomosis to achieve duodeno-ileostomy diversion in adults with severe obesity was feasible and safe, achieved excellent weight loss, and resolved type 2 diabetes at 1-year follow-up.

**Trial Registration:**

Clinicaltrials.gov Identifier: NCT05322122.

**Graphical Abstract:**

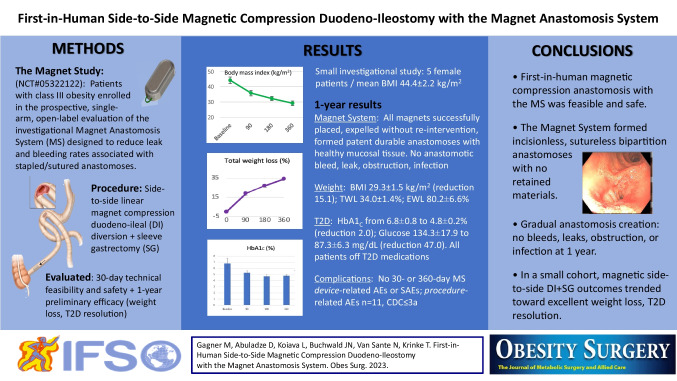

## Introduction

Metabolic/bariatric surgery (MBS) is safe and markedly more effective and durable than medical therapy in attaining weight loss and type 2 diabetes (T2D) resolution [1, 2]. Recently updated guidelines summarizing more than 30 years of evidence broadened the range of individuals for whom MBS is recommended to patients with a body mass index (BMI, kg/m^2^) ≥ 35.0 with or without associated medical conditions (AMCs) and those with a BMI of 30.0–34.9 with metabolic disease [1]. MBS safety and accessibility are also advancing through minimally invasive surgical (MIS) techniques, such as those that require only one anastomosis (e.g., single-anastomosis duodeno-ileostomy with sleeve gastrectomy (SADI-S)), and through technologies that reduce the risks of suturing and stapling (e.g., compression anastomosis (CA) devices) [3, 4].

Safe anastomosis formation has long been a surgical aim. In 1826, Lembert inaugurated a model for enhanced healing and anastomotic patency by suturing the apposed serosal surfaces of small bowel (enterorrhaphy) [5, 6], while in the same year, Denans introduced a metallic ring for sutureless CA that incorporated Lembert’s inverted tissue technique [7]. Though stitching the anastomosis remained the nineteenth-century standard of care, a spring-loaded version of Denans’ CA rings was popularized by Murphy as the anastomosis button (“Murphy’s button”) in 1892 [8]. Efficient creation of anastomoses was furthered by Hultl and Fischer’s 1908 prototype stapler, and by the mid twentieth century, linear and circular staplers were in use to expedite complex and MIS operations [9]. Yet, as with sutures, staple retention introduced sources of potential acute and chronic complication.

Interest in intestinal CA reignited in the 1980s with Kanshin et al.’s AKA-2 colorectal CA device [10]; Hardy et al.’s biofragmentable anastomotic ring (BAR, 1985 [11], the first CA device used in MBS [12]); and Nudelman et al.’s nickel-titanium shape-memory alloy CA ring/clip (NiTi CAR/CAC, 2000) [13]. While CA devices proved safe and effective, particularly in colorectal procedures [14], most required fixation with retained sutures or clips. Several magnetic compression anastomosis (MCA) technologies were next studied in animals [15–22] and in humans, including an “endoscopic gastroenteric anastomosis with magnets” (EGAM) mechanism [23], the “magnamosis” device [24], a samarium-cobalt magnet [25], and “self-forming magnets” (SFM) [26], although none can be placed incisionlessly and/or free of sutures/staples.

Our group developed a novel MCA technology, the magnet anastomosis system (MS), that creates an incisionless, sutureless intestinal anastomosis without retained materials. Healing occurs gradually over several weeks, facilitating optimal collagen deposition in the formation of a robust anastomosis that may markedly lessen bleeding, leak, stricture, and infection. Our study of the prototype MS in animals demonstrated short-term safety and feasibility [27], providing the foundation for this first-in-human (FIH) investigation. The current study evaluated the safety, feasibility, and preliminary efficacy of the MS in creating a side-to-side duodeno-ileostomy (DI) diversion to improve weight loss and glycemic control in patients with obesity and T2D.

## Patients and Methods

### Study Design and Endpoints

The currently reported prospective observational study represents the first stage of a two-part single-arm open-label evaluation of the investigational MS (“The Magnet Study,” Clinicaltrials.gov NCT#05322122). The focus of stage one was small cohort evaluation of MS technical feasibility and safety at 30 days in a single center, as well as ongoing safety and preliminary MBS efficacy through 360 days.

### Ethical Conduct

The protocol met regulatory guidelines governing clinical development of investigational devices and was approved by the medical center’s Ethics Committee. Investigators were required to notify the Ethics Committee of safety events according to the local requirements. All adverse events (AEs) and serious AEs (SAEs) were reviewed by an independent data and safety monitoring board (DSMB) through study end. In accord with the Helsinki Declaration and ISO14155 regulations, 21 CFR Good Clinical Practices, patients’ safety, and well-being were protected.

### *Patients*

Potential study patients identified through existing records were introduced to the aims of the study and its investigational nature, enabling them to make an informed decision regarding participation. Written informed consent was obtained from each patient.

#### Inclusion and Exclusion

Included patients were required to be 18–65 years old with a BMI of ≥ 30.0 to ≤ 50.0 kg/m^2^ and either T2D (HbA1_C_ ≥ 6.5%) and no prior MBS or sleeve gastrectomy (SG) ≥ 12 months previously with T2D with or without weight regain or have a BMI ≥ 40 kg/m^2^ and be interested in undergoing laparoscopic SADI-S where the duodeno-ileostomy (DI) was performed side to side. Patients agreed to refrain for 1 year from additional MBS or reconstructive surgery that would affect body weight; females agreed to forgo pregnancy and use contraception for 1 year. Prescription or over-the-counter weight-loss medication and non-steroidal anti-inflammatory drugs were prohibited 14 days prior to the procedure and during the study course. Included patients agreed to comply with all protocol requirements.

Study exclusions were type 1 diabetes; uncontrolled T2D, hypertension, dyslipidemia, and sleep apnea; use of injectable insulin; prior non-MBS intestinal, colonic, or duodenal surgery; prior trauma, prostheses, disease, scarring, abnormal anatomy, or genetic expressions which prevented or contraindicated the study procedure; refractory gastroesophageal reflux disease (GERD), *Helicobacter pylori* positive, and/or active ulcer disease; large hiatal hernia; inflammatory bowel or colonic diverticulitis; any anomaly precluding orogastric access by gastroscope and catheters; an implantable pacemaker or defibrillator; untreated or poorly controlled psychiatric illness or substance abuse history; pregnancy, breastfeeding, or unwillingness to use an effective contraception method; an AMC that presented a safety concern; a condition contraindicated for laparoscopic access; a surgical or interventional procedure 30 days prior to or after the procedure; stroke/TIA within 6 months prior to study consent; chronic anticoagulation therapy (except aspirin); active infection requiring antibiotic therapy; inability to comply with the follow-up schedule; participation in another clinical investigation; known allergies to the device components or contrast media; limited life expectancy due to terminal disease; a positive COVID-19 test prior to the procedure; and any condition that might preclude follow-up assessment through day 360.

### Device

#### Magnet System (MS)

**Fig. 1:**
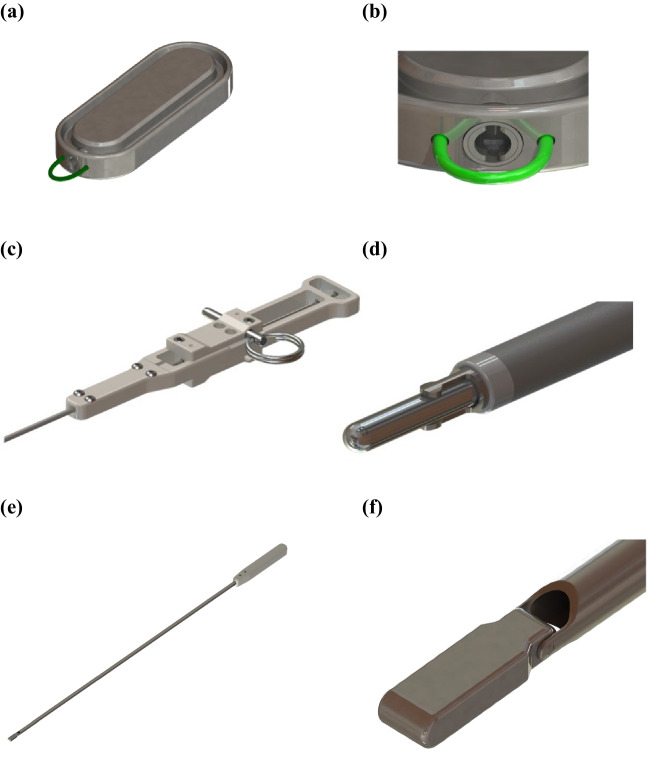
**a** Magnet system (MS) assembly; **b** MS loop attachment feature; **c** magnetic anastomosis delivery system (MADS) handle; **d** MADS distal tip;** e** MADS magnetic positioning device assembly; **f** MADS magnetic positioning device distal tip

The investigational device for creation of a side-to-side duodeno-ileal anastomosis by MS (GT Metabolic Solutions, San Jose, CA) comprises a pair of linear BC42 neodymium magnets (0.75″ length × 0.25″ width × 0.125″ thickness) with a 2.3-mm-offset perimeter flange and Ti-6Al-4 V ELI grade-23 titanium casing (KJ Magnetics, Pipersville, PA) (Fig. 1a). A stainless steel/nitinol/polyester suture loop at one end of each magnet facilitates secondary magnet positioning or retrieval (Fig. 1b).

#### Magnet Anastomosis Delivery System (MADS)

The MADS is a flexible orogastric delivery catheter made of stainless steel/nitinol and Hytrel® polymer (Dupont, Wilmington, DE) used to engage and advance MS magnets intraluminally in the small bowel (Fig. 1c, d). The laparoscopic MS positioning device (GT Metabolic Solutions, San Jose, CA) has a non-magnetic titanium elongate tube and hinge pin (Fig. 1e) with a magnetically attractive stainless steel articulating tip (Fig. 1f) for magnet advancement through the jejunum and ileum.

### Procedure

**Fig. 2:**
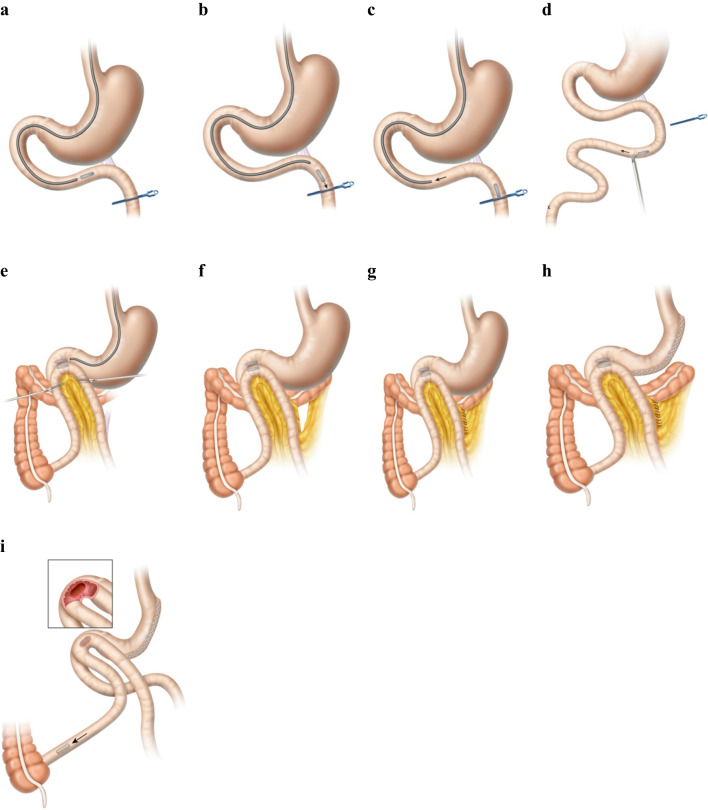
Side-to-side duodeno-ileal magnetic compression anastomosis procedure with the magnet system (MS). **a** After a marker is placed in the ileum 250 cm from ileocecal valve, a retrievable metal bowel clamp is positioned 10–15 cm distal to ligament of Treitz and the first (distal) MS magnet is transported orogastrically to the ligament of Treitz; **b** the distal magnet is released and attracted toward clamp; **c** the endoscope is withdrawn; **d** the bowel clamp is removed and the magnet is directed by the positioning device through the jejunal lumen to the marked position in the ileum; **e** non-magnetic bowel forceps elevate the distal magnet in the ileum over the transverse colon, anterior and latero-lateral to the postpyloric duodenum, and the proximal MS magnet is delivered endoscopically to the duodenal fusion site and released to align with the distal magnet through the intestinal walls; **f** the endoscope and magnet positioning device are withdrawn; the mesenteric defect remains; **g** the mesenteric defect is closed; and **h** a sleeve gastrectomy is performed. **i** In 5–7 days, magnets are fused; 2–6 weeks later, the fused magnet pair detaches from the duodeno-ileal site and is expelled naturally; food flows through the duodenal lumen and also through the patent anastomosis into the ileal lumen

Under general anesthesia, a marker was laparoscopically placed in the ileal mesentery 250 cm from the cecum with medium to large titanium clips, and a retrievable metal bowel clamp (Aesculap AG, Tuttlingen, Germany) was placed 10–15 cm distal to the ligament of Treitz. The first (distal) MS magnet was transported orogastrically by flexible endoscopy (Pediatric 190 colonoscope, Olympus America, Center Valley, PA) to the fourth part of the duodenum and released in the proximal jejunum, where it was attracted toward the clamp. The endoscope was retracted to the level of the stomach and its contents aspirated. A positioning device was used to grasp the magnet, the clamp was removed, and the positioner directed the magnet through the jejunal lumen to the marked ileal position. The distal magnet in the ileum was elevated over the transverse colon with 2 non-magnetic bowel forceps and brought anterior and latero-lateral to the duodenum. The second (proximal) magnet was delivered through the endoscope to the intended magnet fusion site in the first duodenum and released to self-align with the distal magnet through the intestinal walls. The endoscope and magnet positioning device were withdrawn, and the mesenteric defect was closed with running 2–0 silk suture, on its left side (Fig. 2). The SG procedure was then carried out with an endostapler (Ethicon, Cincinnati, OH), a leak test performed, and a left drain placed.

Postoperatively, in 2–4 weeks, the magnets were expected to be fully fused after compressing, necrosing, and sloughing the tissue between them. Subsequently, paired magnets were intended to detach from the duodeno-ileal site and be expelled naturally. Food flows through the duodenal lumen and also through the patent anastomotic diversion into the ileal lumen.

### Postoperative Care

After the procedure until discharge, patients were carefully monitored with attention to hemodynamic conditions and cardiac rhythm. On day 1, successful MS placement was confirmed by abdominal X-ray and fluoroscopically using barium or Gastrografin. Predischarge, patients met with a dietitian or nutritionist to review the postprocedure diet. Patients underwent evaluation within 11–17 days of the procedure, attended 6 follow-up visits (days 30, 60, 90, 180, 270, and 360), and returned for unscheduled office visits as needed.

### Outcome Measures

The primary endpoint was device feasibility at 30 days confirmed by (1) successful technical placement of the MS magnets, (2) expulsion of magnets without device-related AEs requiring surgical reintervention, and (3) creation of a patent duodeno-ileal anastomosis confirmed fluoroscopically and endoscopically. The endpoint was considered met if device performance was confirmed in ≥ 80% of patients. The primary safety endpoint was 30-day incidence of device- and procedure-related AEs (e.g., intra-abdominal hematoma) and SAEs (e.g., SAEs requiring intervention, e.g., intestinal perforation or obstruction, life-threatening bleeding) using the Clavien-Dindo classification [28].

Secondary endpoints were ongoing safety and MBS efficacy measures of change in weight, HbA1_C_, and glucose through a 360-day follow-up. Measurements included absolute weight (kg); total weight loss (%TWL: [initial weight − follow-up weight]/[initial weight] × 100); excess weight loss (%EWL: [initial weight − follow-up weight]/[initial weight – ideal body weight] × 100); BMI loss (initial BMI – postintervention BMI); and the proportion of patients with > 5.0% TWL.

### Statistical Analysis

Descriptive statistics were calculated using the SPSS statistical package (version 20.0; IBM, Chicago, IL). Primary endpoints were represented by categorical variables and reported using frequencies and percentages. Summary statistics for continuous variables were reported using means and standard error of the mean (SEM). Group mean changes in weight and metabolic parameters were assessed using the paired sample *t*-test; alpha was set at *p* < 0.05.

## Results

### Patient Characteristics

On November 22, 24, 25, and 26 of 2021, 5 female Caucasian patients with a mean age of 44.2 ± 7.9 years (range 34–55) underwent side-to-side MS DI followed by an SG. Mean baseline weight was 117.6 ± 7.1 kg (100–140) with a BMI of 44.4 ± 2.2 (37.6–50.8). Four patients (80.0%) had T2D (group mean HbA1_C_ 6.8 ± 0.8%, glucose 134.3 ± 17.9 mg/dL) (Table 1) treated with anti-diabetic medications that were stopped on the day of the procedure; one patient stopped 2 of 3 medications but continued on Glucophage (Merck Santé, Darmstadt, Germany) through day 90.

**Table 1:** Baseline patient demographic and clinical characteristics

Characteristics	*N* = 5
Age, yrs, mean ± SEM (range)	44.2 ± 3.5 (34–55)
Females, *n* (%)	5 (100.0)
Ethnicity, Caucasian	5 (100.0)
Weight, kg, mean ± SEM	117.6 ± 7.1
Body mass index, kg/m^2^, mean ± SEM	44.4 ± 2.2
Associated medical conditions, *n* (%)	
Type 2 diabetes mellitus	4 (80.0)
Non-alcoholic steatosis disease	2 (40.0)
Dyslipidemia	2 (40.0)
Hepatic steatosis	1 (20.0)
HbA1_C_, %, mean ± SEM	6.8 ± 0.8
Glucose, mg/dL, mean ± SEM	134.3 ± 17.9
Prior sleeve gastrectomy ≥ 12 months, *n* (%)	0 (0.0)
Indicated for SADI-S, where duodeno-ileostomy is side to side, *n* (%)	5 (100.0)
Smoking status, *n* (%)	1 (20.0)
Menopause, *n* (%)	3 (60.0)

*HbA1*_*C*_ glycosylated hemoglobin, *SADI-S* single-anastomosis duodeno-ileostomy with sleeve gastrectomy

### Perioperative Course

**Fig. 3:**
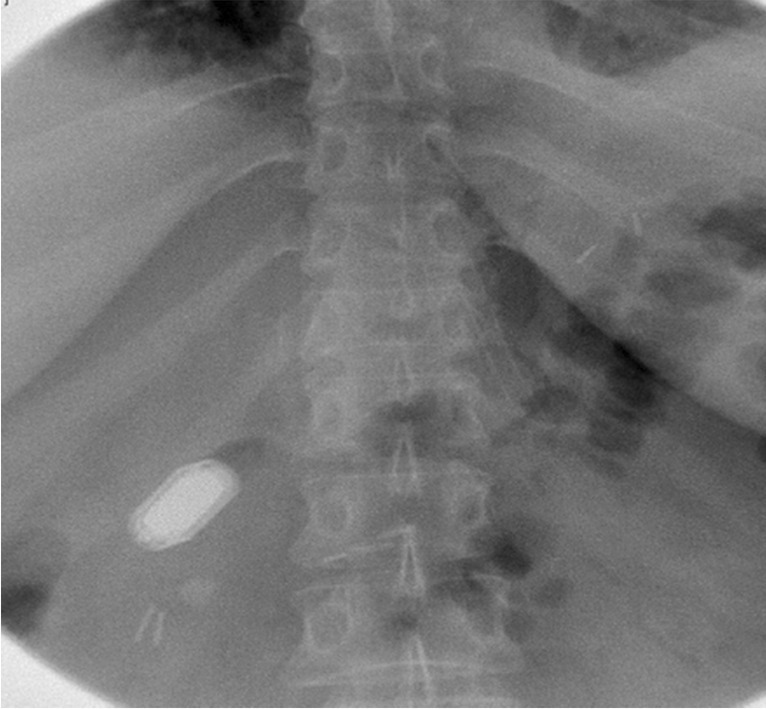
Patient radiograph of magnet alignment and fusion in the right upper quadrant, postoperative day 1. Note the presence of marking clips at 250 cm on the magnet side

Each patient underwent side-to-side MS DI without intraoperative complications. Initially, the MADS laparoscopic positioning device was used to advance the distal magnet to the ileum, but this maneuver was found to be better accomplished using the traction of a single MS magnet directed by laparoscopic forceps. Following successful pairing of the DI magnets (Fig. 3), an SG procedure was performed in the manner of Gagner [29, 30].

Total mean side-to-side MS DI operative time was 138.4 ± 12.4 SEM minutes (median 129.0; min 114.0, max 185.0) including a mean 57.0 ± 15.2 min (median 49.0; 28.0, 115.0) to position the distal magnet at the ligament of Treitz; 60.0 ± 2.7 min (median 59.0; 54.0, 68.0) to direct the magnet to the ileal position; and 13.4 ± 4.7 min (median 10.0; 2.0, 30.0) to appose distal and proximal magnets for fusion. Procedure segment time estimates represent the early operative learning curve and instrumentation. The mean time to magnet expulsion was 58.2 ± 12.5 days.

### Thirty-Day Feasibility and Safety

**Fig. 4:**
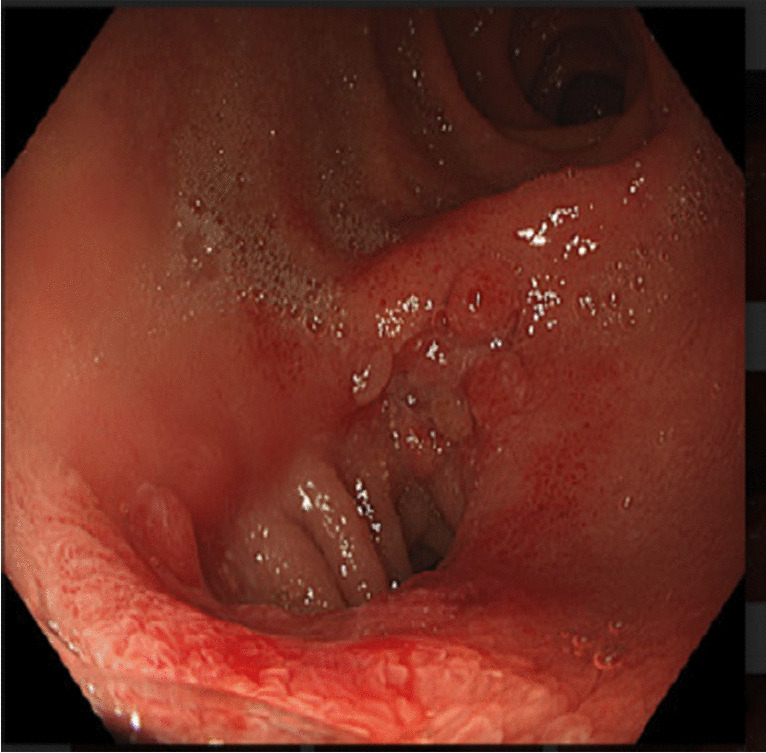
Endoscopic image of a patent side-to-side duodeno-ileal anastomosis with the magnet system. View is from the pylorus toward the first part of the duodenum

The MS was successfully placed in all 5 patients (100.0%) and creation of a patent anastomosis was confirmed radiologically and fluoroscopically in 100.0% of procedures (Fig. 4). All paired magnets were expelled successfully without surgical reintervention. The time to device expulsion (subject to self-report) was 22–92 days (mean 58.2 ± 12.5 days), some taking longer due to individual variability. The primary feasibility endpoint was considered met.

During the first 30 days, there were no device-related AEs or SAEs. Three patients had mild pain in their postoperative abdominal wounds (CDC grade I); these were treated with an intramuscular analgesic injection and resolved without sequelae. One patient had a mild mucosal tear of the upper esophagus due to endoscopic overtube insertion (CDC grade I; use of an overtube was subsequently removed from the procedure); the event resolved on the same day without sequalae. Another patient sustained an intra-abdominal hematoma adjacent to the SG staple line in the upper left quadrant diagnosed by CT scan (CDC grade II); she was observed for 24 h due to poorly controlled hypertension and required no transfusions. The hematoma was on the side opposite from the magnets and adjacent to the sleeve staple line. A final patient had a serosal tear of the ileum due to a pulling motion by laparoscopic forceps during the procedure (CDC grade III); this was immediately sutured as a precaution and resolved on the same day (Table 2).

**Table 2:** Adverse events by number and severity post side-to-side magnet system duodeno-ileostomy with sleeve gastrectomy through day 360 by Clavien-Dindo Classification

	All patients (*N* = 5) *n* (%)					
Adverse event	Grade I	Grade II	Grade III	Grade IV	Grade V	Total
Mucosal tear of upper esophagus due to overtube insertion	1	0	0	0	0	1 (6.3)
Serosal tear of ileum (5 mm) due to laparoscopic forceps	0	0	1	0	0	1 (6.3)
Mild abdominal pain from procedure wounds	3	0	0	0	0	3 (18.8)
Intra-abdominal hematoma at sleeve staple line, upper left quadrant	0	1	0	0	0	1 (6.3)
Vitamin B_12_ deficiency	3	2	0	0	0	5 (31.3)
Vitamin D deficiency	0	1	0	0	0	1 (6.3)
COVID-19 positive	3	0	0	0	0	3 (18.8)
Constipation	0	1	0	0	0	1 (6.3)
Number of adverse events	10 (62.6)	5 (31.2)	1 (6.2)	0 (0)	0 (0)	16 (100)

Clavien-Dindo Classification of surgical complications [28]: grade I, deviation from the normal postoperative course without the need for pharmacological treatment or surgical, endoscopic, and radiological interventions. Antiemetics, antipyretics, analgesics, diuretics, electrolytes, and physiotherapy allowed. Grade II, requiring pharmacological treatment with drugs other than such allowed for grade I complications. Blood transfusions and total parenteral nutrition included. Grade III, requiring surgical, endoscopic, or radiological intervention. Grade IV, life-threatening complication (including certain central nervous system complications) requiring intermediate care/intensive care unit management. Grade V, death of a patient

### Days 31–360

#### Efficacy

**Fig. 5:**
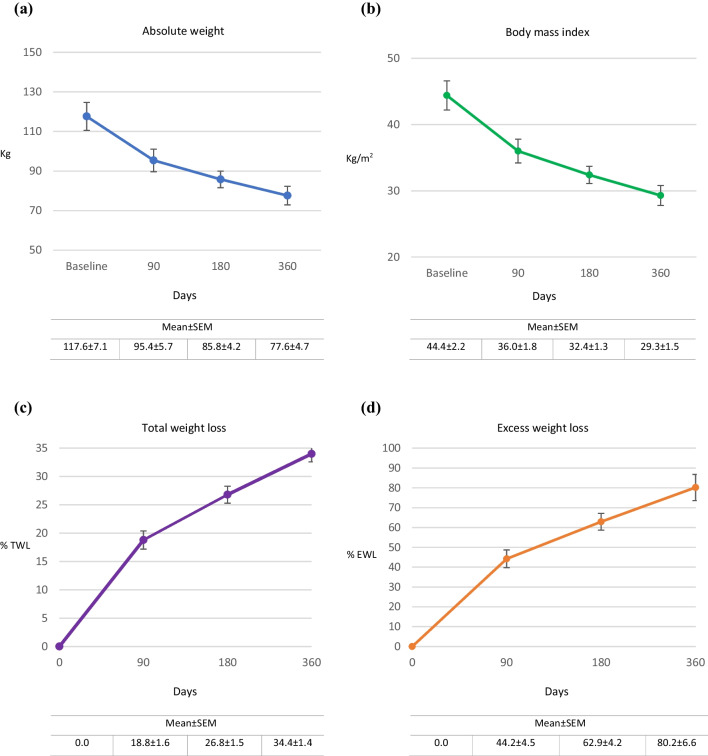
Mean changes in weight from baseline to 90, 180, and 360 days following side-to-side magnet system duodeno-ileostomy with sleeve gastrectomy in **a** absolute weight (kg) and **b** body mass index (BMI, kg/m.^2^) and from 90 to 180 and 360 days in **c** total weight loss (%TWL) and **d** excess weight loss (%EWL)

At 360-day study end, the 4 patients (80.0%) with complete endoscopy results had patent anastomoses with healthy mucosal tissue. Figure 5a and b depicts evolution of mean body weight and BMI for the 5 individual patients at days 90, 180, and 360. Group mean absolute weight fell from 117.6 ± 7.1 kg at baseline to 77.6 ± 4.7 kg at day 360, for an overall mean weight change of 40.0 kg (*p* < 0.001); group mean BMI was reduced from 44.4 ± 2.2 to 29.3 ± 1.5, an overall change of 15.1 (*p* < 0.001). Figure 5c and d depicts corresponding progressive increases in EWL and TWL at days 90, 180, and 360 for each patient. Respective mean EWL and TWL at day 360 were 80.2 ± 6.6% and 34.0 ± 1.4%; 100.0% of patients achieved > 5.0% TWL at 12 months.

**Fig. 6:**
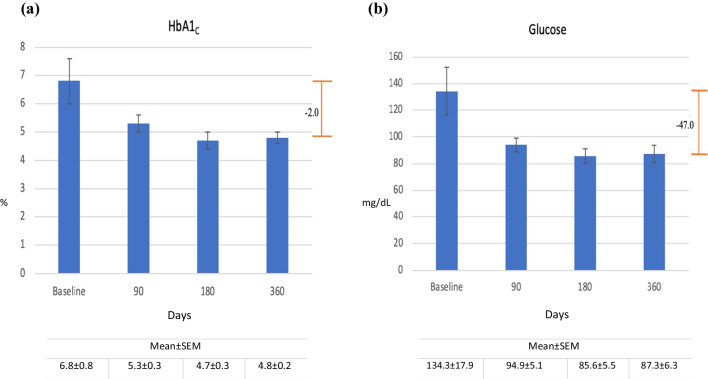
Mean changes in metabolic parameters from baseline to 90, 180, and 360 days following side-to-side magnet system duodeno-ileostomy with sleeve gastrectomy in **a** HbA1_C_ (%), reduction of 2.0%, and **b** blood glucose (mg/dL), reduction of 47.0 mg/dL

The 4 patients with T2D did not take antidiabetic medications or ceased taking them on their procedure day. One patient stopped taking Siofor (Berlin-Chemie, Germany) and Diabetone (Vitabiotics, London, UK), but continued Glucophage through day 90, stopping after experiencing a 1.2% reduction in HbA1_C_. This patient moved out of the country near study end and was not available for the 360-day blood sample. Figure 6a and b depicts HbA1_C_ and glucose changes through day 360. Group mean HbA1_C_ fell from 6.8 ± 0.8% at baseline to 4.8 ± 0.2% at day 360, an overall mean HbA1_C_ change of 2.0 ± 1.6% (*p* = 0.07); group mean glucose was reduced from 134.3 ± 17.9 mg/dL to 87.3 ± 6.3 mg/dL, an overall mean reduction of 47.0 mg/dL (*p* = 0.07).

#### Safety

In continuity with the perioperative period, there were no device-related AEs or SAEs and no mortality through study end. Ten additional procedure-related AEs accrued between day 31 and 360: 3 patients tested positive for COVID-19 which resolved (CDC grade I); 1 patient experienced constipation near day 360 which was effectively treated with standard medication (CDC grade II); 1 patient had a vitamin D deficiency which resolved with supplementation (CDC grade II); and at ≥ 180 days, 5 patients who entered the study with normal vitamin B_12_ levels required B_12_ supplementation which normalized the deficiency (CDC grades I (*n* = 3) and II (*n* = 2)). One B_12_ outcome was unknown as the patient was not available after moving out of the country.

## Discussion

Surgical magnets have been applied to gain exposure, dissect tissue planes, achieve hemostasis, and recently, to create an anastomosis. This first-in-human study was aimed at evaluating the 30-day feasibility and safety of the magnet system in creating a side-to-side duodeno-ileal diversion and at observing preliminary 360-day weight loss and T2D resolution. Our predicate study of the MS device in swine successfully demonstrated that the insertion technique was technically straightforward and that the compressive force of linear magnets mated through duodenal and ileal bowel walls would form a patent anastomosis with an internal diameter of > 20.0 mm. At 6-week postprocedure, gross and histologic examination confirmed well-healed anastomoses with minimal inflammation and good vascularization compared to sutured enterotomy sites. The current human study corroborated these preclinical findings.

All 5 patients (100.0%) met the primary endpoint: magnets were successfully placed and subsequently expelled without surgical re-intervention, and magnetic formation of patent anastomoses comprised of healthy mucosal tissue was attained. There was no mortality nor device-related AEs or SAEs at 30 and 360 days. All secondary endpoints trended toward MBS efficacy objectives. Patients had significant marked weight and BMI loss, > 5.0% TWL, and functional improvement of HbA1_C_ and blood glucose at study end.

At this time, MS 30-day feasibility and safety outcomes can only be compared directly to one other report. Schlottmann et al. described 4 females/4 males with a median BMI of 38.8 who underwent side-to-side DI using the self-forming magnets (SFM) device [26]. As in the current study, all DI magnets were placed successfully, expelled with no device-related AEs, and achieved patent anastomoses. Importantly, while both proximal and distal MS magnets and the proximal SFM magnet were positioned by upper endoscopy, the distal SFM magnet was delivered laparoscopically under fluoroscopic guidance through a 5-mm ileotomy closed with absorbable suture. Thus, MS insertion is fully endoscopic, whereas insertion of one of the 2 SFM magnets by enterotomy introduces the possibility of an immediate leak. One-year efficacy outcomes of these two studies cannot be appropriately compared as the MS DI was studied in combination with an SG.

Contemporary non-magnetic CA devices have been shown comparably effective to conventional suturing and stapling [14, 31, 32]; however, MS technology may provide an effective yet safer alternative. Although the sample of the first MS study was small, its preliminary 1-year safety and efficacy trends can be reasonably compared to SADI-S, an MBS procedure first described by Sanchez-Pernaute et al. (2007) as a measure to reduce the risks and complexity of biliopancreatic diversion/duodenal switch (BPD/DS), recently endorsed by the American Society for Metabolic and Bariatric Surgery (ASMBS, 2020) and the International Federation for the Surgery of Obesity and Metabolic Disorders (IFSO, 2021) [33–36]. SADI-S consists of a postpyloric end-to side stapled or sutured anastomosis 250 cm from the ileocecal junction. It is offered as a standalone procedure for patients with class 3 and 4 obesity with T2D/metabolic syndrome and as a first-stage operation for those with class 4 obesity or high-risk patients [36, 37].

In terms of 30-day morbidity, early registry and systematic review data suggest that SADI-S may be as safe as duodenal switch (DS) with a potentially higher incidence of complications than SG and Roux-en-Y gastric bypass (RYGB) [37, 38]. However, these SG and RYGB conclusions may be misleading as SADI-S patients included in the studies had a disproportionately higher mean body weight, BMI, T2D, and dyslipidemia; randomized controlled trial (RCT) evidence on the question of SADI-S vs RYGB is pending [38, 39]. The 2021 IFSO SADI-S position statement provided systematic review evidence for 4540 patients that suggested a 7.8% rate of serious near-term complications (Clavien-Dindo score ≥ 3b) and an 8.0% reoperation rate. Anastomotic leak and bleeding were the major early complications cited most frequently; longer-term complications included bile reflux, gastroesophageal reflux disease, and nutritional challenges [35]. In the current study of side-to-side MS DI, there were no 30- or 360-day MS device-related AEs or SAEs; procedure-related AEs (*n* = 11) included no anastomotic leak or bleeding and were deemed mild (Clavien-Dindo score ≤ 3a), all subsiding within 24 h of treatment without sequelae. Incidence of serious complications with the MS is expected to be quite low since its insertion is technically less difficult than suture and staple application, requires no enterotomies, leaves no foreign materials in the body, and initiates gradual anastomotic healing.

SADI-S weight loss efficacy is considerable, with mean 12-month TWL ranging from 21.5 to 41.2% and EWL from 61.6 to 102.0% [34, 35, 40]. Current mean side-to-side MS DI + SG TWL of 34.0% and EWL of 80.2% at 360 days were comparable to those reported for SADI-S. While SADI-S conducts food directly to a shortened ileal channel, yielding substantial weight loss, this is sometimes at the expense of long-term nutritional adequacy and anemia. In contrast, side-to-side MS DI in combination with SG resulted in very good weight loss while also preserving the integrity of the major papilla and thereby pancreatic, biliary, and liver function within the duodenal metabolism, potentially contributing to nutritional health. If less weight is lost with side-to-side MS DI + SG than with SADI-S, the procedure may be revised to a full SADI-S or DS.

Type 2 diabetes resolution of 60.0–80.0% after SADI-S has been reported, similar to that of BPD/DS which results in the highest rate of T2D resolution among MBS procedures [35]. Two of the 5 patients who underwent side-to-side MS DI + SG began the study without elevated HbA1_C_ and glucose values, yet all participants’ values improved, and the group mean for both metabolic parameters normalized by study end, with all patients off of T2D medication. The number of patients in this study was too small to provide a definitive indication of weight loss or T2D effectiveness; however, outcomes trended toward efficacy.

### Limitations

The current study’s small size limited the efficacy analysis to reporting preliminary trends and interpretation. Studies with larger samples and comparative cohorts or randomized controlled designs are needed to weigh the potential advantages of the MS device vs sutured or stapled anastomoses in MBS procedures.

## Conclusions

This first-in-human study of the novel incisionless, sutureless magnet anastomosis system demonstrated its safety and feasibility to create a patent side-to-side duodeno-ileal anastomosis diversion. Trends toward desired weight loss and T2D resolution were observed at 1-year follow-up.
